# A Phone in a Basket Looks Like a Knife in a Cup: Role-Filler Independence in Visual Processing

**DOI:** 10.1162/opmi_a_00146

**Published:** 2024-06-12

**Authors:** Alon Hafri, Michael F. Bonner, Barbara Landau, Chaz Firestone

**Affiliations:** Department of Linguistics and Cognitive Science, University of Delaware; Department of Cognitive Science, Johns Hopkins University; Department of Psychological and Brain Sciences, Johns Hopkins University

**Keywords:** relations, abstraction, scene perception, intuitive physics, compositionality, language of thought

## Abstract

When a piece of fruit is in a bowl, and the bowl is on a table, we appreciate not only the individual objects and their features, but also the relations *containment* and *support*, which abstract away from the particular objects involved. Independent representation of roles (e.g., containers vs. supporters) and “fillers” of those roles (e.g., bowls vs. cups, tables vs. chairs) is a core principle of language and higher-level reasoning. But does such role-filler independence also arise in automatic visual processing? Here, we show that it does, by exploring a surprising error that such independence can produce. In four experiments, participants saw a stream of images containing different objects arranged in force-dynamic relations—e.g., a phone contained in a basket, a marker resting on a garbage can, or a knife sitting in a cup. Participants had to respond to a single target image (e.g., a phone in a basket) within a stream of distractors presented under time constraints. Surprisingly, even though participants completed this task quickly and accurately, they false-alarmed more often to images matching the target’s relational category than to those that did not—even when those images involved completely different objects. In other words, participants searching for a phone in a basket were more likely to mistakenly respond to a knife in a cup than to a marker on a garbage can. Follow-up experiments ruled out strategic responses and also controlled for various confounding image features. We suggest that visual processing represents relations abstractly, in ways that separate roles from fillers.

## INTRODUCTION

What kinds of properties do we perceive? An intuitive and influential answer to this question is traditionally the one given by David Marr (1982), who famously defined perception as “the process of discovering from images what is present in the world, and where it is” —transforming the light reaching our eyes into representations of objects and their features, located somewhere in space.

**Figure 1.**
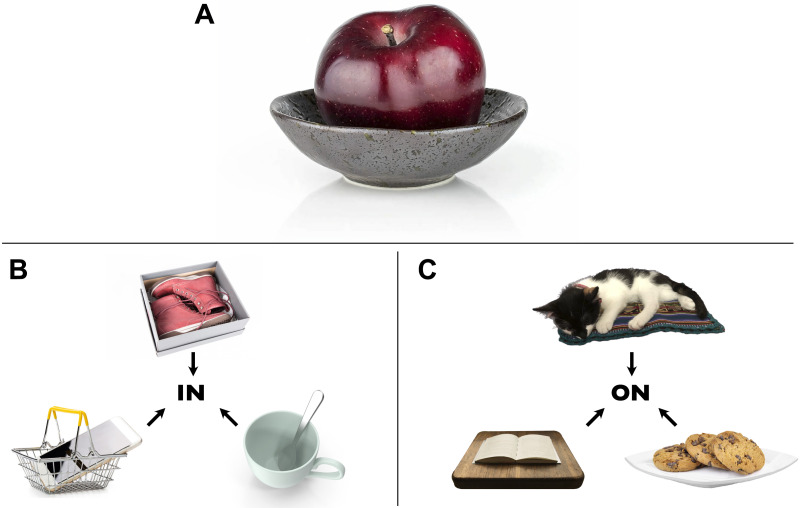
Scenes and situations that evoke the relations Containment (in) and Support (on).^1^ We encounter many everyday instances of these relations, such as fruit in a bowl, and a bowl on a surface (*Panel A*). Yet we can appreciate other instances of these relations—including both familiar and unfamiliar instances (*Panels B and C*)—and these images also appear to instantiate the relations of containment and support.

But is this all that perception delivers? Consider the image in Figure 1A; what do you see in it? Certainly you see some objects and their locations—some reddish fruit in the center, a gray bowl farther down the image, and so on. However, beyond the features (“what”) and locations (“where”) of these objects, you may also see something about *how* the objects relate to one another: The fruit is contained *in* the bowl; the bowl is resting *on* a surface. What is the nature of this experience? And what role does visual processing play in furnishing such representations?

### Representing Relations

Relational representations are a major research focus in psychology, in ways that go far beyond the sorts of between-object relations present in Figure 1. For example, they play a central role in analogical reasoning (as when we generalize relations from one domain to another; Gattis, 2004; Goldwater & Gentner, 2015; Jamrozik & Gentner, 2015), linguistic reference (as when we use or acquire terms like “in”, “on”, “above”, “below”, and so on; Johannes et al., 2016; Landau & Jackendoff, 1993; Levinson, 2003; Quinn, 2007; Talmy, 1983), and even causal ascription (as when we work out whether moving one object caused another to fall; Gerstenberg et al., 2017; Kominsky et al., 2017; Wolff & Song, 2003).

This empirical and theoretical work points to certain essential characteristics of relational representations (e.g., Jackendoff, 1990; Marcus, 2001; Markman & Gentner, 1993; Miller & Johnson-Laird, 1976; among many others). First, relations require *relata*: they hold between entities. For example, one object can be below another; but an object cannot simply be *below*, period, without reference to some other object. Second, relations are *structured*: the “order” of the relata matters. For example, a cat on a mat is very different from a mat on a cat, even though both cases involve the same objects (cat and mat) and the same relation (support); the objects’ *roles*, figure (the located object) and reference (or “ground” object), are reversed in these two situations. Third, relations are *abstract*: they go beyond any one particular instance. For example, fruit may be in a bowl, shoes may be in a box, a phone may be in a basket, and a spoon may be in a mug—and all are equally valid instantiations of in, even though they involve completely different objects that differ in size, shape, color, texture, and more (Figure 1B). The same holds for other relations, such as support or on (Figure 1C).

### “Role-Filler Independence” Is Core to Relational Representation

The aforementioned characteristics of relations together make for a representational scheme that is often called *role-filler independence*: the capacity to bind representations of arbitrary entities (the fillers, or relata) to distinct roles, in ways that preserve the identities of both the fillers themselves (e.g., cat and mat) and the abstract relation (e.g., on and its corresponding roles, figure and reference; Frankland & Greene, 2020; Hummel & Holyoak, 2003; Quilty-Dunn et al., 2022). This representational format is especially flexible and powerful; indeed, it has often been assumed that higher-level cognitive processes like language and analogical reasoning must implement (or approximate) role-filler independence in order to achieve the systematicity, productivity, and compositionality they characteristically exhibit—for example, the capacity to understand sentences one has never heard before, or to reason about how entities relate in novel ways (Fodor, 1975; Fodor & Pylyshyn, 1988; Holyoak & Lu, 2021; Marcus, 2001).

Prior work in domains such as analogical reasoning has found that people can appreciate abstract relational structure from visual images (e.g., the similarities between a man giving a woman food and a woman feeding a squirrel; Gattis, 2004; Goldwater & Gentner, 2015; Goldwater et al., 2011; Markman & Gentner, 1993). These studies have revealed much about how humans generate metaphors, analogies, or similarity judgments across stimuli (see also Goldstone, 1994a, 1994b; Hahn et al., 2003). Indeed, the work in this domain provides considerable empirical and theoretical support for the existence of role-filler independence in at least some higher-level cognitive processes (Holyoak & Lu, 2021; Hummel & Holyoak, 2003).

### Our Question: Role-Filler Independence in Visual Processing Itself?

Despite the extensive work on relational representations in higher-level cognitive domains, it remains unclear what role visual processing plays in generating relational representations in the mind. Of course, any task involving a visual stimulus trivially implicates visual processing at *some* level (at minimum, in extracting basic visual properties such as colors, textures, or edges)—but crucially, this does not mean that visual processing itself also implements role-filler independence. Notably, prior work has thus far used methods that are not well suited for answering this question; for example, asking participants to give ratings on a scale of 1 to 9 on how well two pictures match (Markman & Gentner, 1993) or to choose which of two sentences best matches an image (Gattis, 2004). Although such methods implicate relational processing in general, they cannot implicate the telltale signatures of visual perception that we explore further below, such as rapidity or automaticity (for a review of such signatures, see Hafri & Firestone, 2021, and Scholl & Gao, 2013).

Consider Figure 1A again. One may perceive colors, textures, locations, and even perhaps the categories of the objects in the image (e.g., a shiny piece of fruit, or a gray bowl). This does not mean that the relation in the image is itself perceived; observers may see more basic visual properties and subsequently *reason* or *judge* (via more deliberative cognitive processes) that the apple must be in the bowl on the basis of those properties. Indeed, it is reasonable to think that this might be the case: in studies on analogical reasoning, participants often must deliberate intently before arriving at judgments of similarity (e.g., Markman & Gentner, 2000; Ratcliff & McKoon, 1989), suggesting that it is possible that extraction of abstract relational structure from visual stimuli happens primarily or only at the level of conceptual (and/or linguistic) processing.

Alternatively, perhaps visual perception itself generates representations that are both relational and abstract. Intriguingly, recent evidence suggests that surprisingly sophisticated visual relations between objects—such as chase, cause, and socially interact—show telltale signatures of automatic visual processing, such as being extracted rapidly and spontaneously, and in ways that influence other visual processes (Chen & Scholl, 2016; Firestone & Scholl, 2016, 2017; Guan & Firestone, 2020; Hafri et al., 2013, 2018; Kominsky & Scholl, 2020; Little & Firestone, 2021; Papeo & Abassi, 2019; Papeo et al., 2017; Rolfs et al., 2013; for a review, see Hafri & Firestone, 2021; for work on relations within objects, e.g., between object-parts, see Barenholtz & Tarr, 2006; Biederman, 1987; De Winter & Wagemans, 2006; Feldman & Singh, 2006; Firestone & Scholl, 2014; Hummel & Stankiewicz, 1996; Palmer, 1978).

Crucially, however, none of this work has asked whether visual processing respects the core relational property of role-filler independence, i.e., whether it maintains the identity of certain relations apart from their participating entities. A positive answer to this question would shed new light not only on mechanisms of relational representation itself, but also on the kinds of contents perception can represent in the first place, and the formats used to represent them—perhaps not only iconic, analog, or “picture-like” (Block, 2023; Carey, 2009; Kosslyn et al., 2006) but also discrete, symbolic, or “sentence-like” (Hafri et al., 2023; Mandelbaum et al., 2022; Quilty-Dunn, 2020; Quilty-Dunn et al., 2022)—a point to which we return in the General Discussion.

### The Present Experiments: “Confusing” Instances of the Same Relation

Here, we investigated whether role-filler independence arises in visual processing by looking for a hallmark of its abstract nature: generalization from one relational instance to another. In particular, we asked whether the similarity of otherwise very different relational instances is powerful enough that those instances may be *confused* for one another, even when they involve completely different objects and visual features (such as those in Figures 1B and 1C). If such relational confusions were observed in a time-constrained visual task that does not require attending to the relation itself, this would provide evidence that the visual system automatically processes relations in ways that abstract away from the particular objects involved—in other words, role-filler independence. This would be evidence that abstract relational representations arise not only in explicit and deliberate judgments, but even in tasks that do not require such reasoning (and even discourage it).

As a case study, we investigated a pair of force-dynamic relations: containment and support (e.g., phone in basket, knife in cup, spoon on box, shovel on garbage can). This class of relations encompass physical forces between objects; as such, they are central to many other cognitive processes and domains, such as scene perception (Biederman et al., 1982; Võ & Henderson, 2009), language (Bowerman, 1996; Landau, 2017; Levinson & Wilkins, 2006; Vandeloise, 2010), cognitive development (Baillargeon et al., 2012; Casasola et al., 2003; Hespos & Spelke, 2004), and intuitive physics (Davis et al., 2017). Furthermore, recent modeling work has suggested that certain visual cues (e.g., systematic differences in border ownership between objects) might reliably indicate the presence of containment or support regardless of the particular objects involved—even from static images (Ullman et al., 2019). Thus, these relations are ideal for exploring whether visual processing implements role-filler independence.

To explore relational confusions, we created an image set of various household objects participating in containment and support relations of the sort depicted in Figure 1. We then asked participants to perform a straightforward visual recognition task: to respond to a pre-specified target image (e.g., phone-in-basket) embedded in a continuous stream of non-target images, all presented in a time-constrained manner. Crucially, when specifying the target image, we made no mention of “in,” “on,” or any other relational properties, nor did our task itself require encoding such properties. Nevertheless, we reasoned that if the same abstract property in is evoked even from very different scenes that instantiate this property, then under time pressure, the mind might be prone to confusing one example of in for another—and that this would manifest in increased false-alarms for images that matched the relational category of the target image (relative to images that did not). In other words, we predicted that participants who were looking for a phone in a basket might be more likely to mistakenly respond to a knife in a cup than to a spoon on a box (Experiment 1)—even as they continued to represent the objects themselves. Furthermore, they should show such relational confusions even in cases where performing the task based on the participating objects alone would be totally sufficient (Experiment 2). Finally, we predicted that such confusions would go beyond the lower-level correlates of these relations (Experiments 3a and 3b). If so, these results would suggest that the visual system furnishes relational representations in ways that abstract away from the particular objects involved in the relation—a case of role-filler independence in visual processing.

## EXPERIMENT 1—RELATIONAL CONFUSIONS

Might observers confuse one instance of a relation with another in a time-constrained visual task? Experiment 1 showed participants photographs of different household objects participating in relationships of Containment (in) and Support (on) and asked them to identify a target image among non-target images. Despite the simplicity of the task, we predicted that observers would false-alarm more when non-target images matched the target’s relational category, even with very different objects.

### Methods

#### Participants.

For this study, 200 participants were recruited through Amazon Mechanical Turk (for discussion of this pool’s reliability, see Crump et al., 2013, who replicate several core findings from cognitive psychology on this platform). This sample size was chosen because it is similar to that of other studies with a similar design and participant pool (e.g., Guan & Firestone, 2020). The advertisement for the study specified that they should only participate if they were at least 18 years of age and were native speakers of English, and the consent form required them to click a checkbox affirming that they met these conditions of participation.

#### Stimuli.

To create the stimulus set for this experiment, we took photographs of everyday objects in an indoor environment, rendered in grayscale. In each image, one of 11 object pairs was depicted in one of two force-dynamic relations: containment (in) and support-from-below (on). Each pair included unique objects (e.g., Object Pair 1 was knife and cup, Object Pair 2 was candle and bowl, etc.). In Containment (in) images, the smaller object (the *figure*) rested in the larger object (the *reference* object); in Support (on) images, the figure object rested on top of the reference object. It has been proposed that these kinds of containment and support are the “core” or central subtypes of these relations. This proposal is supported by two observations: (i) linguistic expressions for these subtypes are generally among the earliest acquired relative to other more “extended” subtypes (e.g., a crack embedded *in* a mug, or a suit hanging *on* a hook; Landau et al., 2016; Landau, 2018); and (ii) these subtypes are hypothesized to be present across all languages, often marked by the simplest expressions in a language (Levinson & Wilkins, 2006). (We return to the distinction between core and non-core subtypes in the section titled From Language to Vision of the General Discussion.)

**Figure 2.**
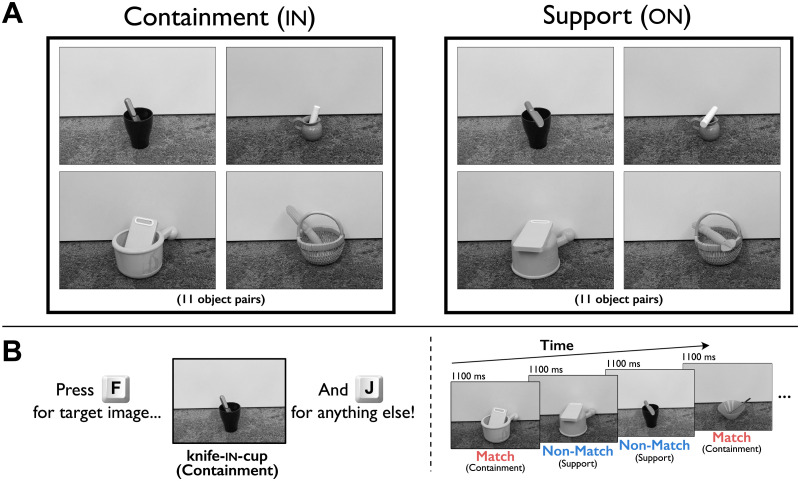
Stimuli and task. *Panel A*: Eleven different object pairs appeared in both Containment (in) and Support (on) images. *Panel B*: In the task, participants had to press one key for a pre-specified target image, and another key for every other image (a fixation cross [200 msec] and then a blank frame [100 msec] also appeared before each image in the trial sequence but are not depicted, for simplicity). Crucially, half of the non-target images *matched* the relation category of the target image (here, in), and half did not. In Experiment 2, the image depicting the alternate object pair of the target was not included. For example, if the target image was *knife in cup*, the participant never observed the *knife on cup* image. This was done to eliminate the possibility that including this alternate image induced a strategy of attending to the relations depicted in the images.

Importantly, all object pairs participated in both relations; see Figure 2A for example images. Each image was 800 × 600 pixels in size. (Due to the nature of online experiments, we cannot specify here the exact size, viewing distance, brightness (etc.) of the images as they appeared to participants, because we could not know each participant’s particular viewing conditions. However, any distortions introduced by a given participant’s viewing distance or monitor settings would have been equated across all stimuli and conditions.)

#### Procedure.

There were two epochs (or halves) of the experiment, each featuring a different target image. In one epoch the target was a Containment (in) image and in the other it was a Support (on) image, with each target image depicted by different object pairs (selected at random from the 22 stimulus images). Epoch order and the object pairs for each target image were randomized across participants. For example, for Participant 1, target images for the first and second epochs may have been Object-Pair 3 in Containment (in) and Object-Pair 7 in Support (on), respectively; for Participant 2, they may have been Object-Pair 4 in Containment (in) and Object-Pair 5 in Support (on); and so on.

During the instruction phase before each epoch, participants were shown their target image and were told to find the target image among a sequence of non-target images. They were then informed about their task: to press one key (F) for their target image and another key (J) for any other image as fast and accurately as possible. Importantly, nothing in the instructions mentioned anything about containment, support, or other relational properties. Participants were informed that all images (target and non-target) could appear in their original orientation or mirror-flipped horizontally (randomly from trial-to-trial), to make the task more difficult. Participants were instructed to respond appropriately to each image regardless of its orientation.

During the experimental task, images appeared in a continuous stream, one after the other (1,100 msec each). Each image was preceded first by a fixation cross (200 msec) and then by a blank frame (100 msec). Participants received feedback on each trial: upon keypress, the image border turned green for correct responses, and red for incorrect responses or failing to respond within one second. The purpose of this feedback was to keep participants attentive to the task, as we found in piloting that participants would become disengaged without it.

There were 192 trials in total across the entire experiment (96 per epoch). Within each epoch, there were four blocks of trials. Each block contained trials with all images except the target image from the other epoch: the target image (repeated four times) and the 20 non-target images. Trial order was randomized within-block, and trials appeared in a continuous sequence one after another within epoch (i.e. there was no break between blocks of trials, only epochs).

Readers can experience the task for themselves at https://www.perceptionresearch.org/abstractRelations/E1.

### Exclusions

Exclusion criteria were consistent across experiments (and were pre-registered in later experiments). First, to ensure that the included participants were likely to have remembered the pre-specified target image, we excluded epochs if 50% or fewer of the 16 target image instances were correctly identified (implying that the participant forgot what the target image looked like). This exclusion criterion applied without regard to the nature of non-target responses.

Second, to ensure that the included participants followed the task instructions and performed reasonably well on the main task (distinguishing target from non-target images), we excluded participants with less than 80% accuracy (on trials used for the main analysis only; see below), frequent timeouts (on more than 25% of trials), or implausibly fast responses (RTs < 100 msec on more than 15% of trials).

Finally, to ensure that display timing in participants’ browsers met the intended precision, we excluded participants with a high degree of display timing imprecision (on more than 5% of trials), i.e. the measured durations of the fixation, blank, or trial image deviated from the expected durations by more than 33 msec (corresponding to two frames at a frame rate of 60 Hz), or the average browser frame rate for the trial was less than 30 Hz or more than 140 Hz (as measured by the performance.now javascript method). After excluding participants for these various reasons, we excluded any remaining individual trials with display timing issues (0.2%).

A total of 31 participants (16% of the recruited sample) were excluded by these criteria. However, we note that no result reported here or in later experiments depended in any way on these exclusions; in other words, all of the results reported below remained statistically significant, in the same direction, even when we include all participants and trials.

### Analysis

Our primary question in this study concerned *generalization*: In particular, we expected to observe more false-alarms to non-target images that matched the target’s relational category, even when such images had completely *different* objects and visual features. Thus for our main analyses, we excluded non-target images that depicted the same object-pair as the target (e.g., if the target was knife-*on*-cup, we excluded knife-*in*-cup from analyses). (Indeed, in Experiment 2, we removed such images entirely, to ask whether we would observe an effect of relational confusion even when extracting the relation is not useful for the task.)

We tested our predictions formally with mixed-effects logistic regression on trial-level data (analyzing non-target trials only). Mixed-effects models allow for generalization of statistical inferences simultaneously across participants and items by accounting for both participant- and item-level variability, even without an equal number of trials in each condition (Baayen et al., 2008; Barr et al., 2013). The dependent variable was accuracy. The independent variable of interest was Match Type: whether the image matched the target’s relation or not, sum-coded. The independent variables Target Category (in or on, sum-coded) and Epoch Number (centered) were also included as main effects and interaction terms with the main variable of interest (Match Type), in case the effect varied by the target’s relational category or changed from the first to the second epoch.^2^ We tested for significance of variables by using likelihood-ratio tests on the Chi-square values from nested model comparisons with the same random effects structure (Baayen et al., 2008). We expected a significant effect of Match Type: that is, when the trial image matched the relation depicted in the target image, participants would be more likely to false-alarm, even when the image depicted a different set of objects.^3^

### Results and Discussion

As expected, participants responded quickly (mean RT = 499 msec), and performance on the main task (i.e., target and non-target discrimination) was quite high, at 96%. Accuracy was also higher on non-target trials (98%) than on target trials (84%). (This may be explained by the lower prevalence of target trials, which were rare and usually required changing one’s response from the previous trial.) Thus, the task was relatively easy for participants to perform successfully, as was intended.

**Figure 3.**
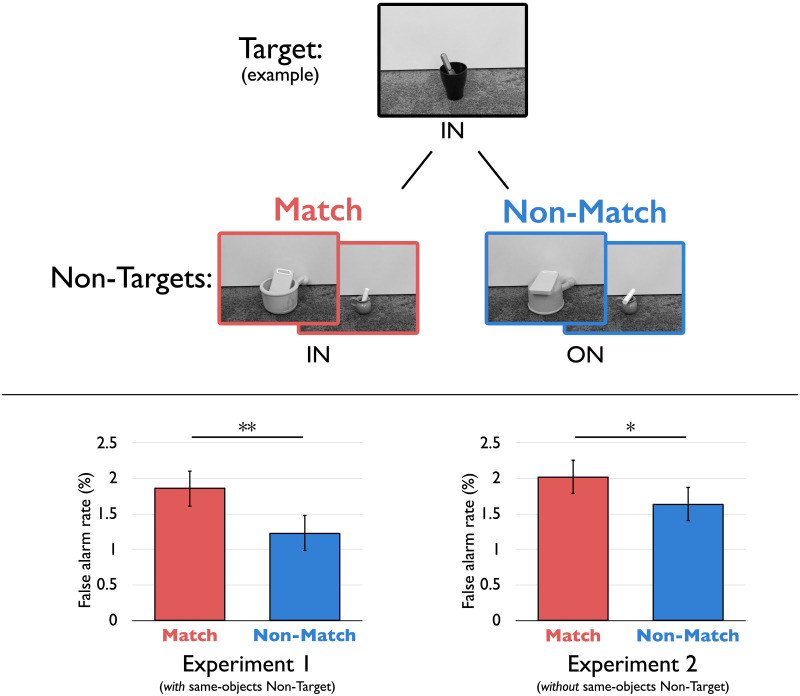
Results from Experiments 1 and 2, showing spontaneous extraction of force-dynamic relations (in and on) from natural scenes in a manner that generalized across different objects. In Experiment 1 (bottom-left panel), participants false-alarmed more often to non-target images that matched the target’s relational category (red bars) than those that did not (blue bars), even though the objects themselves differed. This was true even in Experiment 2 (bottom-right panel), where extracting the relation was not necessary for performing the task because the object pair in the target image never appeared as a non-target image (e.g., if knife-in-cup was the target, knife-*on*-cup never appeared). Error bars depict within-participant 95% confidence intervals. ** *p* = .006, * *p* = .01.

However, participants occasionally made errors. Remarkably, as can be seen in Figure 3 (bottom-left panel), when participants did make such errors, they false-alarmed more often for non-target images that matched the target’s relational category than for those that did not—even though such images contained very different objects and visual features.

These conclusions were confirmed using mixed-effects logistic regression.^4^ The best fitting model was one that included a main effect of Match Type (match vs. non-match), as compared to an otherwise identical model without this factor, *χ*^2^(1) = 7.65, *p* = .006. Adding interactions of Match Type with Target Category or Epoch Number did not significantly improve the fit (both *χ*^2^’s < 1.34, *p*’s > .246), suggesting that the Match Type effect was similar across categories (in and on), and throughout the duration of the experiment (thus suggesting that participants did not develop a relational matching strategy from the first half of the study to the second).

Moreover, participants were also slower to correctly reject non-target images when these images matched the force-dynamic relational category of the target (based on comparison of linear mixed-effects models on reciprocal response time data with and without Match Type as a factor, *χ*^2^(1) = 9.70, *p* = .002). This implies that it took longer for participants to “overcome” the relational information to provide a correct answer on these trials. Put differently, when searching for a phone in a basket, participants took longer to say that knife-in-cup was not their target than to say that knife-on-cup was not their target. However, this effect, though robust and reliable, was not the main prediction of interest, so we do not discuss it further.

To summarize, this first experiment suggests that participants extracted the force-dynamic relations of the images they were observing in a manner abstracted away from the particular identities of the objects involved. In other words, when looking for a phone in a basket, participants mistook it for a knife in a cup.

#### Mechanisms Underlying the Relational-Confusion Effects: Visual or Cognitive?

Note that our task differs from traditional investigations into how the mind represents the similarity of two or more stimuli (a set of processes that often fall under terms such as analogical mapping or structural alignment; Gattis, 2004; Goldstone, 1994a, 1994b; Goldwater & Gentner, 2015; Goldwater et al., 2011; Hahn et al., 2003). In those tasks, participants engage in explicit and deliberate reasoning about the degree to which certain stimuli are similar. Indeed, it has been suggested that this reasoning process can be computationally intensive, such that more time may be needed to represent and compare stimuli with underlying relational similarities than more superficial similarities (Markman & Gentner, 1993; Ratcliff & McKoon, 1989). By contrast, here participants were engaged in a time-constrained visual recognition task: identifying the exact target image, and only that image, before the trial timed out. This task did not require participants to make explicit judgments of the kind usually featured in the analogical reasoning literature (e.g., similarity ratings). Even so, participants in our task could not help but process the images relationally (and as discussed in Experiment 2 below, this was true even in cases where extracting the objects alone was totally sufficient for performing the task).

#### Role-Filler Independence, or Just Minimal Representation of Fillers?

Abstracting visual relations away from the participating entities is an important prerequisite for role-filler independence in visual processing. However, role-filler independence also requires that the fillers for the roles in the relations (e.g., the objects acting as figure and reference in on) be independently represented. Although we interpret the above effects as reflecting such role-filler independence, an alternative explanation is a kind of “abstraction by impoverishment,” whereby perception represents relations by stripping out most object content (colors, shapes, categories, etc.) from relational representations, laying bare “empty” object files represented in some particular spatial configuration (for articulation of such a view, see Hochmann & Papeo, 2021). One version of this explanation might posit that filler objects are simply represented elsewhere in the perceptual system. However, an extreme form of this “impoverishment” explanation would be that participants in our task were representing fillers only minimally, if at all, and that this was the source of the image confusions we observed. Such an outcome would not provide evidence for role-filler independence in visual processing, contrary to what we have been suggesting.

Opposing the more extreme form of this abstraction-by-impoverishment explanation, certain patterns in our data suggest that observers were in fact representing fillers in our task. First, the nature of our experimental design allows us to ask whether there is not only a relation-confusion effect, but also an object-confusion effect: Observers false-alarmed for non-target images with the same objects as the target on 41.67% of such trials on average, as compared to only 1.23% of trials for images with different objects (excluding same-relation non-target images), *χ*^2^(1) = 57.83, *p* < .001.^5^ For example, if their target was an image with a knife in a cup, they false-alarmed more often for an image with a knife on a cup than a pencil on a bowl. This would only occur if participants were representing some aspect of the stimuli beyond the relation. Moreover, we also tested for any relationship between this object-confusion effect and the relational-confusion effect across observers. In particular, if some observers were representing filler objects more than other observers were, the abstraction-by-impoverishment account would predict that those observers who extracted fillers less well (marked by a smaller object-confusion effect) would also confuse images of the same relation with one another more often (i.e., a negative correlation). To test this, we first applied the empirical-logit transform to each participant’s same-object and same-relation false-alarms, which normalizes the probability space by increasing the variance of values near floor and ceiling. Notably, after correlating these values across observers, we found that the two effects were actually *positively* correlated (*r*(167) = 0.25, *p* < .001)—exactly opposite the direction that abstraction-by-impoverishment would predict, or at least the strong form of it in which filler objects are only minimally represented. Instead, this positive correlation between same-object and same-relation false-alarms is likely reflective of general, overall performance on the task.

Thus, we took the present results as compelling initial evidence for role-filler independence in visual processing.

## EXPERIMENT 2—TRULY SPONTANEOUS?

Experiment 1 suggested that the mind extracts representations of force-dynamic relations in the course of automatic visual processing. However, it is possible that the nature of the experimental design encouraged participants to encode relational category as a strategy to perform the target identification task. In particular, given that one of the non-target images contained the very same objects as the target image (e.g., when the target image was a knife in a cup, there was also a knife on a cup as a non-target image), participants may have discovered that it was useful to attend to the relation in each image to perform the task. But if the task itself makes extracting relational category a helpful strategy, then this extraction would not quite be spontaneous or automatic. We did not find evidence in Experiment 1 that participants adopted such a strategy, as the relational-confusion effect was not statistically different between epochs of the experiment. Nevertheless, a stronger test of our central hypothesis would make the relation depicted in the target image *totally irrelevant* for performing the target identification task.

Experiment 2 did just that, by simply omitting the non-target images whose objects corresponded to those in the target image. For example, if a participant was asked to respond to a knife *in* a cup, they were never shown a knife *on* a cup as a non-target. Thus, extracting relations was not necessary, and indeed extracting object categories alone was totally sufficient; in other words, participants could rely solely on the (arguably more prominent) object category information—or indeed, on any number of lower-level properties such as shape, size, or shading of these objects—to be successful on the task. If we still observe the relational-confusion effect here, it would be even stronger evidence for the automatic or spontaneous nature of the effect.

### Methods

#### Participants.

225 participants were recruited through Amazon Mechanical Turk for this study. (This sample size was larger than for Experiment 1 in order to equate the raw number of non-target trials per participant to be analyzed across the experiments, i.e. 144 here vs. 160 in Experiment 1). Conditions for participation (i.e., age and speaking English as a native language) and exclusion criteria were the same as in Experiment 1. A total of 29 participants (13% of the total) were excluded by the criteria.

#### Stimuli and Procedure.

Experiment 2 was identical to Experiment 1, apart from the following changes. The main change was that alternate object-pair images (i.e. non-target images that had the same object pairs as the target images) were completely excluded from a given participant’s session. For example, if Participant 1 had Object-Pair 3 as their Containment (in) target and Object-Pair 7 as their Support (on) target, then Object-Pair 3 in Support (on) and Object-Pair 7 in Containment (in) never appeared for that participant. Thus, in each experimental epoch, there were 19 unique images (one target image repeated 4 times per block, and 18 non-targets), with 176 trials in the experiment in total.

Readers can experience the task for themselves at https://www.perceptionresearch.org/abstractRelations/E2.

### Results and Discussion

As expected, participants responded quickly (mean RT = 492 msec), and performance on the main task (i.e., target and non-target image discrimination) was quite high, at 96%. Accuracy was again higher for non-target trials (98%) than for target trials (88%). Crucially, as can be seen in Figure 3 (bottom-right panel), participants once again false-alarmed more often for non-target images that matched the target’s relational category than for those that did not —even when extracting relational category was completely irrelevant to the task.

These conclusions were again confirmed using mixed-effects logistic regression.^6^ The best fitting model was one that included a main effect of Match Type (match vs. non-match), as compared to an otherwise identical model without this factor, *χ*^2^(1) = 6.38, *p* = .01. Adding interactions of Match Type with Target Category or Epoch Number did not significantly improve the fit (all *χ*^2^’s < 0.25, *p*’s > .61), suggesting that the Match Type effect was similar across categories (in and on), and throughout the duration of the experiment (again suggesting a minimal role for development of a relational strategy over the course of the study).

To summarize, we still observed confusions between spatial relations involving totally different objects, even when encoding such relations on each trial was not obviously useful for the target detection task (i.e., when the task could have been performed based only on the objects in the scene). Thus, these results suggest that the extraction of abstract force-dynamic relations is not dependent on specific strategies that might be useful in this task; rather, it appears to happen spontaneously upon observation of a visual scene.

#### Does Explicit Awareness of the Relational Categories Matter?

A crucial aspect of our study design was that the instructions made no mention of relations, containment, support, “in-ness,” “on-ness,” and the like; participants were simply told to remember their target image. However, an important question is whether they may have nonetheless become explicitly aware of these relational categories, and whether this awareness led to the relational-confusion effects we observed.

To explore this question, we examined the post-experiment questionnaire, which included an open-ended comments box as well as the following question: *“In this experiment, there were several categories of interest (groups of images that were related in a certain way). If you had to guess, what might have been the categories?”*. This allowed us to test quite directly whether the participants explicitly noticed the in or on categories. We checked whether participants mentioned a word indicative of at least one of the two relational categories (for in: one object being “in”, “inside of”, or “contained by” another; for on: one object being “on”, “outside of”, “out of”, or “across” another). To the degree that explicit awareness of the relational categories did not predict our effect of interest, then the claim that relations were encoded spontaneously would be strengthened.

We found that only a minority of participants even mentioned in or on when prompted to guess about the categories they saw (20% in Experiment 1, and 15% in Experiment 2). This suggests that by and large, they did not seem to find these categories especially notable, at least in their explicit reports. (Instead, most participants reported categories such as “kitchen items,” “squares and circles,” “cups, bowls, baskets,” “I have no idea,” etc.).

Crucially, we also found that explicit awareness of the categories in and on did not predict the size of the relational-confusion effects observed, as confirmed by additional analyses. We fit mixed-effects logistic regression models for Experiments 1 and 2, introducing the binary variable Relation-Mention (sum-coded) into the corresponding best-fitting models (which included the key predictor Match Type, i.e., Match vs. Non-Match to the target’s relational category). In Experiment 1, we observed a main effect of Relation-Mention (*χ*^2^(1) = 5.12, *p* = .024), indicating that participants mentioning in or on showed more false-alarms overall. Crucially, however, this factor did *not* interact with Match Type (*χ*^2^(1) = 0.86, *p* = .355), indicating that explicit awareness of relational categories did not statistically increase the likelihood of the relational-confusion effect. The same is true for Experiment 2, which showed no significant effect of Relation-Mention (*χ*^2^(1) = 2.33, *p* = .127), nor its interaction with Match Type (*χ*^2^(1) = 1.11, *p* = .292).

Furthermore, the key effect of Match Type remained significant even after totally excluding relation-mentioners, despite the inherently lower power (Experiment 1: *χ*^2^(1) = 6.26, *p* = .012; Experiment 2: *χ*^2^(1) = 4.24, *p* = .040). Qualitatively similar results were obtained when broadening the criteria for what counted as mentioning in or on to include any mention of “containers.”

In summary, when participants were explicitly asked about the categories after the experiment, only a small minority reported in or on, and those who did mention such relations did not exhibit a stronger relational-confusion effect. While it is possible that additional participants were aware of these relations without explicitly mentioning them, these analyses tentatively suggest that explicit awareness of relations did not significantly impact the degree of spontaneous encoding of relations during the study.

#### Mere Differences in Amount of Occlusion?

An alternative explanation for our results focuses on a crucial property that varied among images in our stimulus set: the amount of occlusion of the Figure object. Containment and support relations differ in this property, and it even varies within instances of containment. For example, in Figure 2, the pencil in the bowl is barely occluded, while almost half of the knife is occluded in the cup. If the target was an in image, it is possible that participants were more likely to false-alarm to a test image with greater Figure occlusion (and vice versa for an on target).

First, it is important to note that this explanation does not distinguish between graded occlusion as a *visual cue* to categorical in or on relations (Halberda, 2019) and a graded representation itself. We can nevertheless explore the role of continuous occlusion in our task’s performance. If continuous occlusion does not predict relational-confusion effects, it strengthens our confidence that participants represented images in terms of categorical relations.

To achieve this, we used photo editing software to manually select the visible area of the Figure object in each object-pair image (e.g., knife in cup, pencil on bowl) and the inferred whole area of the Figure object, including the occluded portion. We calculated the proportion of the Figure object occluded by the Reference object (in pixels). We then incorporated this continuous Proportion-of-Occlusion predictor (empirical-logit-transformed) into the best-fitting mixed-effects logistic regression models for Experiments 1 and 2, alongside the key predictor Match Type (Match vs. Non-Match).

We first tested one straightforward prediction (unrelated to the relational-confusion effect itself): that increased occlusion would make it more challenging to discern the object’s identity, decreasing overall accuracy (i.e., increasing overall false-alarms). This was confirmed: it was significant in Experiment 1 (*χ*^2^(1) = 7.06, *p* = .008) and trending in Experiment 2 (*χ*^2^(1) = 2.58, *p* = .108). This suggests that our occlusion measurements were reasonable.

For the key test of the relationship between continuous occlusion and relational-confusion effects, we introduced the (logit-transformed) predictor “Proportion-of-Occlusion-*Match*”: for in targets, this was the proportion of the Figure object that was occluded (zero for most on test images); for on targets, this was the proportion of the object that was visible (1.0 for most on test images). This variable aims to capture the prediction that if the amount of occlusion matching the target’s relational category matters, we should observe a positive relationship between occlusion and false-alarm rate in the in condition and a negative relationship in the on condition. However, this variable did not significantly improve model fit in either Experiment 1 (*χ*^2^(1) = 0.07, *p* = .786) or Experiment 2 (*χ*^2^(1) = 0.52, *p* = .470). This remained true even when only analyzing trials where the test image’s category was in, suggesting that this lack of relationship was not simply due to the largely uniform nature of the Proportion-of-Occlusion-Match variable in on test images. While these exploratory analyses must be interpreted with caution, as they may not have sufficient power to detect these particular effects, they do suggest that at minimum, the categorical coding of each trial image (as in versus on) “does the job,” sufficiently capturing the distinction we are targeting.^7^

## EXPERIMENT 3—RELATIONAL CATEGORIES PER SE

Although the images in Figures 1 and 2 naturally evoke impressions of force-dynamic relations, it is possible that the image-confusions we observed in Experiments 1 and 2 were driven not by these relations themselves, but rather by lower-level image properties that happen to correlate with these higher-level relations. While exploratory analyses in the section titled Mere Differences in Amount of Occlusion? suggest that differences in the amount of occlusion of the Figure object played little role in these effects, other low-level differences between in and on images remain. For example, many of the images depicting containment involve more vertical edges than the images depicting support (due to the contained object resting vertically in the container), or produce differently shaped contours where the two objects meet. Although lower-level features such as these likely contribute to generating impressions of relations (much as curved contours contribute to generating a face percept; Halberda, 2019), higher-level relations go beyond these lower-level features. For example, relational representations require representing not only that certain edges or contours are present but also that two distinct entities (i.e., relata) are in some configuration with one another.

To rule out lower-level explanations of the relational-confusion results, we conducted two additional experiments that used two very different (but complementary) types of distorted control stimuli, described below. Crucially, these distortions eliminated the subjective impression of one object being contained or supported by another, while preserving many other image properties. We predicted that, with these manipulations, the relation-specific image confusions previously observed would disappear, suggesting that it was the relational categories in and on per se, rather than confounded image features, driving these effects.

**Figure 4.**
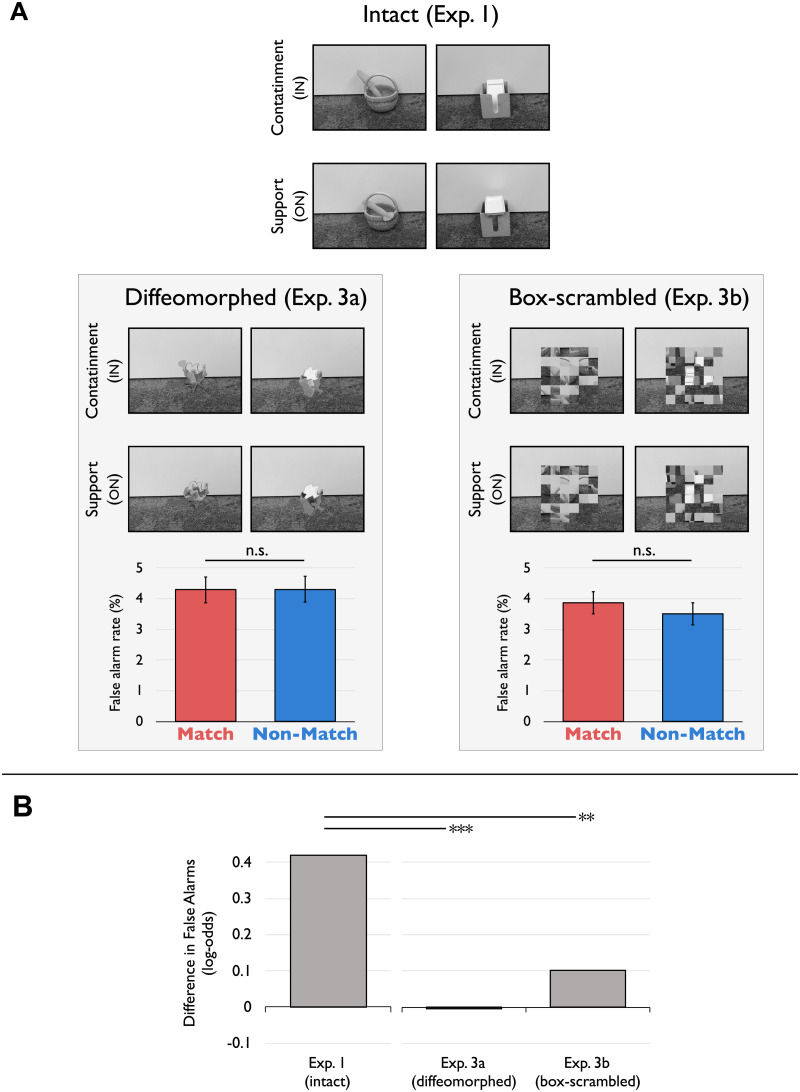
*Panel A*: In Experiments 3a and 3b, images were diffeomorphed and box-scrambled, respectively, to control for lower-level visual features in the original images that might have covaried with relational category. Examples of these images are shown here, along with their intact image counterparts. Unlike in Experiment 1, a relational-confusion effect (more false-alarms to images matching the relation of the target than not matching) was not observed in Experiments 3a and 3b. Error bars depict within-participant 95% confidence intervals. *n.s.* (*p*’s > .33). *Panel B*: The matching false-alarm effect was also significantly higher in Experiment 1 than in 3a and 3b (depicted is the difference of the mean false-alarms between Match and Non-Match across participants, in log-odds space). These results demonstrate that the effect is not due to simple lower-level visual features correlated with relations. *** *p* < .001, ** *p* < .01.

Both studies proceeded identically to Experiment 1, with the only exception being that the images used (both target and non-target) were distorted versions of the original images. In the first control study, Experiment 3a, we applied a diffeomorphic transformation to the objects in the image (Stojanoski & Cusack, 2014). This transformation preserves the percept of a coherent shape contour and some lower-level features such as color and size, while simultaneously rendering the objects unrecognizable. This is achieved by expanding and contracting the image as if it were on a rubber sheet, using flow fields made up of 2D cosine components. (For a more detailed explanation of this image manipulation technique, including code for applying such manipulations, see Stojanoski & Cusack, 2014). In the second control study, Experiment 3b, we box-scrambled the images. This technique randomizes square patches of the image such that local image features are preserved but the percept of coherent visual objects is eliminated. Examples of each transformation can be seen in Figure 4. (Note that these manipulations have advantages over other ways of controlling for lower-level differences, including image inversion. Though inversion would preserve lower-level features, such inverted images may still be recognized as the relations in and on; indeed, the reader may still get such relational impressions when flipping this manuscript upside-down and glancing at Figures 1 and 2.)

These two manipulations thus provided complementary control of lower-level aspects of the stimuli, in that they preserve different aspects of the intact relational images. The diffeomorphic transformation preserves the percept of a coherent object contour in a way that the box-scrambling does not; doing so may be important, as coherent objects are a necessary component of relations. By contrast, the box-scrambling preserves local image features more veridically than the diffeomorphic transformation (Freud et al., 2017). In both cases, the subjective impression of a force-dynamic relation is far less apparent, or even eliminated completely (as our results below confirm).

### Methods

#### Participants.

For these two studies, the hypotheses, sample sizes, exclusion criteria, and an analysis plan were all pre-registered. 200 participants were recruited through Amazon Mechanical Turk for each experiment (i.e., 400 participants in total), to match the sample size of Experiment 1. A pilot version of this task suggested an exclusion rate of approximately 37%, which was not surprising given that both target and non-target images were distorted, making the task more difficult in general. Based on power analysis of the data from Experiment 1, we determined that with this sample size, after exclusions of this rate, we would have approximately 92% power to detect an effect of the same size of Experiment 1 or greater. Conditions for participation and exclusion criteria were the same as in Experiments 1 and 2. A total of 73 participants (37% of the total) in Experiment 3a and 76 participants (38% of the total) were excluded in Experiment 3b.

#### Stimuli.

Examples of each image type can be seen in Figure 4. To create the diffeomorphed images, we first cropped, padded, and removed the background of the images. These cropped images were then diffeomorphed, which involves applying a flow field generated from 2D cosine components with random phase and amplitude. Put more simply, different parts of each image were randomly expanded and contracted stepwise, as if the image were a rubber sheet. The level of diffeomorphing used was the same level at which recognition of similar object types was significantly reduced in previous work (level 12 in Stojanoski & Cusack, 2014). After the cropped images were diffeomorphed, they were each superimposed on a background image, in their original locations in the scene. We created 20 sets of diffeomorphed images. The same random seed was used for all images within a set.

To create the box-scrambled images, we divided the region of each image centered on the objects of interest into a 6 × 6 region of “boxes” 68 × 68 pixels each (with the size of the region corresponding to the largest square extent that fully encompassed all object pairs). We then scrambled the location of the boxes in each image, with the constraint that no box be in the same relative location to another box as it was in the intact image, horizontally, vertically, or diagonally. As with the diffeomorphed images, the box-scrambled image region was superimposed on a background image, in its original locations in the scene. We created 20 sets of box-scrambled images, each with a different randomization. The scrambling locations of boxes were the same for all images within a set.

#### Procedure.

The design and procedure of Experiments 3a and 3b were identical to Experiment 1. All images (both target and non-target) were distorted, in each experiment. Importantly, like in Experiment 1 (but unlike in Experiment 2), the alternate object-pair images (i.e. non-target images from the same object pair as the target images) *were* included in the image sequences. We chose this design precisely because it should be more likely to show an effect; thus, if it did *not* show an effect, that failure would be all the more conclusive. As in Experiment 1, in each experimental epoch (half), there were 21 unique images (one target repeated 4 times per block, and 20 non-targets), and there were 192 trials in total. In each experiment, each participant was randomly assigned one of the image sets (i.e., a set with a different random seed for image creation), without replacement.

Readers can experience the two tasks for themselves at https://www.perceptionresearch.org/abstractRelations/E3a (diffeomorphs) and https://www.perceptionresearch.org/abstractRelations/E3b (box-scrambling).

### Results and Discussion

Participants responded quickly (3a: 520 msec; 3b: 541 msec), and performance on the main task (target and non-target image discrimination) was again high (3a: 93%; 3b: 93%). As before, accuracy was also higher on non-target trials (3a: 96%; 3b: 96%) than on target trials (3a: 83%; 3b: 79%). Crucially, these distorted control manipulations had the intended effect of reducing or even eliminating the relational-confusion effect. This can be seen in two ways. First, participants in both experiments false-alarmed at similar rates for non-target images generated from images that matched the target’s relational category as for those that did not (Figure 4a, bottom-left and bottom-right panels). Second, the effect in Experiment 1 (with intact images) was much stronger than the effect in either of these distorted control experiments (Figure 4b), suggesting that any contribution of lower-level image properties to the relational-confusion effects observed in our earlier experiments cannot be attributed solely to the lower-level differences between the intact relational images for in and on.

These two conclusions were confirmed using mixed-effects logistic regression.^8^ First, we tested for evidence of our earlier relational-confusion effect in the two control experiments. For Experiment 3a (diffeomorphs), a model with a main effect of Match Type (Match vs. Non-Match) was not a significantly better fit than a simpler model without, *χ*^2^(1) = 0.00002, *p* > .99. Adding interactions of Match Type with Target Category (in or on) or Epoch Number to a model with a main effect of Match Type did not significantly improve the fit over a model without the main effect of Match Type (all *χ*^2^’s < 0.14, *p*’s > .93).

For Experiment 3b (box-scrambling), the results were similar: A model with a main effect of Match Type was not a statistically better fit than a simpler model without, *χ*^2^(1) = 0.96, *p* = .33. A model with a main effect and interaction of Match Type and Target Category was marginally better than one with just a main effect of Match Type, *χ*^2^(1) = 3.17, *p* = .08, but this model was not significantly better than a simpler model without any effect of Match Type, *χ*^2^(2) = 4.13, *p* = .12. Adding an interaction of Match Type with Epoch Number to a model with a main effect of Match Type did not significantly improve the fit over a model without a main effect of Match Type, *χ*^2^(2) = 2.30, *p* = .32. Thus, the remaining lower-level differences in these control images were not sufficient to elicit significant relational-confusion effects of the kind observed in Experiment 1 with intact relational images.^9^

Second, we directly tested the difference in the confusion effect across experiments. We ran additional mixed-effects logistic regression analyses comparing the effect of Match Type between Experiment 1 and each control experiment, separately. Indeed, for both control experiments, including an interaction of Experiment by Match Type was a significant improvement over a model with only the main effects of Experiment and Match Type but no interaction: Experiment 3a (diffeomorphs): *χ*^2^(1) = 11.0, *p* = .0009; Experiment 3b (box-scrambling): *χ*^2^(1) = 6.79, *p* = .009.^10^ Although this analysis was only exploratory, it suggests not only that no relational-confusion effects emerged in Experiments 3a and 3b, but also that the significantly more powerful relational-confusion effects observed in Experiment 1 cannot be attributed to the residual lower-level properties that remained in the distorted images used in Experiments 3a and 3b.

Taken together, these results suggest that the image confusion results of earlier experiments were due to spontaneous extraction of abstract relations per se, and not merely the lower-level features correlated with relations (whether the lower-level content present within a globally coherent shape, as in the diffeomorphs of Experiment 3a, or within local image features, as in the box scrambling of Experiment 3b).

## GENERAL DISCUSSION

Does visual processing automatically extract relations in ways that separate roles from fillers? Our experiments suggest that it does, at least for the force-dynamic relations we investigated here. While searching under time pressure for an image of a knife in a cup (for example), participants were liable to confuse that image with other instances of in, even when those instances involved completely different objects (such as a pencil in a bowl, or chalk in a pitcher; Experiment 1). These results held even when extracting the relation was not in any way necessary to complete the task (Experiment 2), and they could not be explained by various lower-level image features (Experiments 3a and 3b). Taken together, these results suggest that when we observe the world, we extract not only the colors, shapes, and locations of the objects around us, but also how those objects *relate* to one another.

Our findings are broadly consistent with recent work demonstrating that perceptual processing of some types of relations is rapid, automatic, and influences other perceptual processes (e.g., motion perception and object detection; Chen & Scholl, 2016; Glanemann et al., 2016; Guan & Firestone, 2020; Hafri et al., 2013, 2018; Kominsky & Scholl, 2020; Little & Firestone, 2021; Papeo et al., 2017; Papeo & Abassi, 2019; Rolfs et al., 2013; Strickland & Scholl, 2015; Vestner et al., 2019; Yuan et al., 2016; for a review, see Hafri & Firestone, 2021). Our results, however, extend these ideas in an important way: We show that this perceptual processing generalizes away from the particular objects involved, in ways that create genuinely *abstract* representations of relations. Across very different instances such as a knife in a cup, a phone in a basket, or a piece of chalk in a pitcher, we see a *commonality*—namely, the relation Containment (in).

### More Than Just Statistical Regularities: Role-Filler Independence and the Format of Visual Representations

This work is related to, but quite distinct from, other work in visual cognition that explores the extraction of visual regularities in space and time (e.g., Fiser & Aslin, 2005; Schapiro et al., 2013). Such work has found that observers learn statistical associations between items over the course of experience, which—as discussed in the literature on *scene grammar*—can afford advantages in perceptual processing of objects, their typical locations, and their relations with one another (e.g., where airplanes generally appear, or where mirrors appear relative to dressers; Kaiser et al., 2014, 2015, 2019; Võ, 2021; Võ et al., 2019; see also Bonner & Epstein, 2021; Kim & Biederman, 2011). Although the mechanisms for learning such regularities are often assumed to be quite general, the associated advantages are usually stimulus- or category-specific: learning that shoes appear in boxes does not afford much information about what things appear on other things in general (e.g., flowers in vases). By contrast, the kind of regularities we have investigated here *are* general, holding over arbitrary instances of relations: Just as we can recognize a pair of shoes in a box or a spoon in a mug (Figure 1B), we can also recognize a phone in a basket—and crucially, we can appreciate that all three are instances of in. The same is true for other relations such as on (Figure 1C).

Notably, our results suggest that visual processing not only represents abstract relations (e.g., in or on), but also their filler objects (e.g., knife and cup), and thus exhibits genuine role-filler independence. Indeed, a supplementary analysis of Experiment 1 (see above section, Mechanisms Underlying the Relational-Confusion Effects: Visual or Cognitive?) showed that observers not only made relational-confusion errors, but they also made confusion errors between images that had the same *objects* appearing in different relations (e.g., a knife-in-cup and knife-on-cup image). This argues against an alternative in which visual processing represents relations by simply discarding information about the objects involved.

The existence of role-filler independence in visual processing dovetails nicely with recent proposals that visual perception instantiates core properties of a “Language of Thought” (LoT; Quilty-Dunn et al., 2022, building on earlier work by Fodor, 1975). A common view is that the format of visual representation is exclusively iconic, or “picture-like” (Block, 2023; Carey, 2009; Kosslyn et al., 2006). Yet the sort of visual representations implied by our findings may be difficult to capture with a purely iconic format, whereby each “part” of the representation corresponds to some part of the represented image. Even a scheme in which abstract relations are represented in a purely object-based manner—i.e., as properties bound to specific objects, with roles like figure or reference represented alongside other properties like color, size, category, etc.—would have trouble accounting for how false-alarms could occur *across* varied objects and scenes (Hochmann & Papeo, 2021). Indeed, in our task, observers treated images with very different objects and visual features (different “parts”) as similar when those objects instantiated the same relation (a property which has no straightforward “part” in the image at all).

Instead, our findings are perhaps more easily accommodated by an abstract, structured representational format with discrete symbols for relations and their filler objects, in the manner Quilty-Dunn et al. suggest. In this way, visual processing may have an important and powerful property in common with certain forms of linguistic processing, namely, its compositional nature—containing discrete constituents that are combined in systematic (and often novel) ways. Just as the compositionality of language supports your ability to understand sentences you have never heard before (Chomsky, 1957; Jackendoff, 1987), and the abstractness of thought supports your ability to generate new thoughts (Fodor & Pylyshyn, 1988), the abstractness of relational perception may permit you to perceive instances of relations you have never seen before (for further discussion, see Hafri et al., 2023).

An open question concerns the degree to which visual relations are represented in a *fully* abstract manner, completely inert to changes in visual context (including to the participating objects). In our studies, we used objects that differed greatly in appearance (e.g., knife, phone, cup, basket, etc.) but all were common household objects. Thus, it is an open question whether the relational representations we observed are *completely* general, holding over totally unfamiliar combinations of objects (even novel ones like “Greebles”; Gauthier & Tarr, 1997). If relational confusions were observed even in these cases, it would strengthen the evidence for full independence of roles and fillers in visual processing. (Notably, role-filler independence does not entail that any arbitrary object may fill any role in any relation. There may still be certain constraints on the participating entities, such as the geometric properties required of the reference object in a containment, i.e., that it has an interior; Landau & Jackendoff, 1993; Talmy, 1983.)

### From Language to Vision

Our use of force-dynamic relations here was inspired by work in developmental psychology and psycholinguistics exploring what infants understand about such relations, and how children and adults come to talk about them (Baillargeon et al., 2012; Casasola et al., 2003; Hespos & Spelke, 2004; Johannes et al., 2016; Landau & Jackendoff, 1993; Levinson, 2003; Quinn, 2007; Strickland & Chemla, 2018; Talmy, 1983). More generally, our results are consistent with a broad and intriguing conjecture that visual processing privileges the same sorts of categories that young infants are sensitive to and that are carved up similarly across languages—such as core notions of objecthood, causality, and events (Carey, 2009; Spelke & Kinzler, 2007; Strickland, 2017). Indeed, there appears to be an intriguing overlap between the kinds of representations found in core-knowledge systems and those that show signatures of visual processing in adults (including being fast, spontaneous, and dependent on subtle stimulus parameters), with strikingly similar patterns of performance and error across the two. For example, the same cohesion violations that surprise infants also cause adults to lose track of an object they are attending to (Huntley-Fenner et al., 2002; vanMarle & Scholl, 2003), and the same event-types that infants encode as similar or different (e.g., containment vs. occlusion) also drive low-level detection performance in adult vision tasks (Strickland & Scholl, 2015). Likewise, work in cognitive development and linguistics suggests that Containment (in) and Support (on) are privileged relational categories that the mind represents early in development and into adulthood, and are thus particularly good candidates for also being represented via automatic perceptual processing.

First, prelinguistic infants identify these force-dynamic relations surprisingly early (by five months of age; Baillargeon et al., 2012; Hespos & Baillargeon, 2006; Hespos & Spelke, 2004) and represent them in a way that generalizes to novel objects (Casasola et al., 2003). Second, evidence from cross-linguistic studies suggests that the two kinds of support and containment we investigated here—one object supporting another from below, and one object resting in another—are “core” subtypes of more general force-dynamic relations (Landau, 2018; Landau et al., 2016). Indeed, there are certain foci or areas of alignment across languages in the basic linguistic terms used for containment and support, and they tend to be centered on these core subtypes (Carstensen et al., 2019). Moreover, the basic terms for containment and support (“in” and “on” in English) are mapped to these core subtypes first before being extended to less canonical subtypes (e.g., interlocking, embedding, or hanging; Lakusta et al., 2020; Landau et al., 2016)—a process which may require functional knowledge about certain objects (e.g. a coat on a hook) or about non-intuitive physical forces such as adhesion (e.g. a stamp on an envelope; Landau, 2017, 2018). The present results complement these developmental and cross-linguistic findings by showing that core subtypes of in and on are not only represented in cognition (in both infants and adults), but are also automatically extracted in visual processing. More generally, our results add to the growing evidence for abstract, categorical information shared by processes in development, language, and perception (Cavanagh, 2021; Hafri et al., 2023; Quilty-Dunn, 2020; Strickland, 2017).

One detail to note is that we only recruited participants who reported being native speakers of English. We did so because it is known that languages differ in how they package spatial relational information (Bowerman, 1996; Carstensen et al., 2019; Landau et al., 2016; Levinson, 2003; Levinson & Wilkins, 2006). Nevertheless, our study opens up new avenues for testing how language experience in general (and experience with specific languages in particular) interact with visual processing of relations. For example, future work could use our task to test speakers of languages such as Dutch that package information about spatial scenes in other ways, differentiating between, say, a laptop on a desk (“*op*”), and a mirror on a wall (“*aan*”) (Carstensen et al., 2019). Likewise, perhaps speakers of languages that mark certain types of containment as special (e.g., for Korean, tight-fitting containment rather than more general containment) would show more specific “relational-confusions” (or other perceptual effects) according to the particular type of containment (Landau et al., 2016; Levinson, 2003; Norbury et al., 2008; Guan et al., 2020; but see Landau et al., 2024). We suspect that in time-limited visual tasks such as ours, such differences in how languages package information about relations will have minimal impact on how they are visually processed, but this is an empirical question.

### Linguistic, Visual, or Conceptual?

Given the influence from psycholinguistics on our approach, one may wonder whether our results might actually be explained by linguistic or conceptual representations, rather than visual ones. Performing our task—one of visual recognition—involves a comparison of the currently perceived test item to memory for the target, which requires some common format. Is this common format visual, or rather is it conceptual or even linguistic? For example, perhaps participants explicitly labeled the target and distractor stimuli (e.g., “My target is *knife-in-cup*”), and it was the overlap in linguistic labels (“in”) rather than genuine *visual* confusions that produced the observed relational-confusion effects. Or, perhaps participants explicitly reasoned about the similarity of the target and each trial image relationally (e.g., performing structural alignment between the representations of the images; Gattis, 2004; Goldwater & Gentner, 2015; Goldwater et al., 2011; Markman & Gentner, 1993)—a deliberate, cognitive process.

One reason to doubt this alternative is the time pressure in our task, which makes an explicitly linguistic explanation (or a deliberate cognitive comparison strategy) less likely. Trials followed one right after another, and on each one, participants were forced to respond rapidly lest the trial time out; indeed, participants typically responded within half a second—likely too little time to linguistically encode each stimulus in succession, or to reason about the stimuli in a way that would produce our observed pattern of results.

Another possibility is that the comparison is made at a conceptual level that is neither linguistic nor visual in nature. This would be consistent with work demonstrating that meaning can be extracted extremely quickly from a visual scene (and is immune to visual masking but not conceptual masking; Potter, 1976). The idea here would be that the participant, with a conceptual representation for the target in mind, would produce a conceptual representation of the test image, and then compare the two. However, here too our evidence tells against this explanation. As reported in the section titled Does Explicit Awareness of the Relational Categories Matter?, explicit awareness of the categories in and on (as reported post-experiment) did not predict the size of the relational-confusion effects observed; furthermore, our effects remained significant even in participants who never mentioned these categories at all.

Finally, the fact that our task engages visual-recognition processes suggests that the observed effects reside at the interface of perception and memory. Thus, the degree to which role-filler independence exists in visual perception, in visual memory, or in both may not be fully decided by our results. Follow-up work could strengthen the evidence that our effects are visual-perceptual rather than linguistic or conceptual by probing for early signatures of visual target identification that arise before linguistic labels for those items may be activated (i.e., starting at about 200 ms; Indefrey, 2011; Indefrey & Levelt, 2004; Morgan et al., 2008)—whether neural correlates (e.g., certain event-related potentials; Fabre-Thorpe et al., 2001) or behavioral ones (e.g., the attentional blink; Shapiro et al., 1997).^11^

### in, on, and Beyond

Though here we explored in and on as case studies of the broader phenomenon of abstract relational perception, there may well be other visual relations that are processed in this way. As noted above, cognitive development and cross-linguistic comparisons may offer clues toward this end (Carey, 2009; Spelke & Kinzler, 2007; Strickland, 2017), as there is intriguing overlap between the patterns and performance and errors that are found in infant core-knowledge studies and those that show signatures of visual processing in adults. One pattern this work points to is that non-social relations require some kind of physical contact over a short spatio-temporal timescale in order to be automatically processed visually (e.g. in causal launching; Kominsky & Scholl, 2020; Kominsky et al., 2017; Leslie & Keeble, 1987; Muentener & Carey, 2010). Indeed, the lack of contact in spatial relations such as above and next to may perhaps be why successfully extracting them requires that certain more effortful visual routines be actively engaged (Franconeri et al., 2012; Holcombe et al., 2011; Ullman, 1984; Yuan et al., 2016). By contrast, physical contact is not necessary to automatically extract many categories of social interaction, although they do still require reliable social-intentional grouping cues (e.g., two bodies facing one another; Goupil et al., 2022; Hafri et al., 2013, 2018; Papeo, 2020; Papeo et al., 2017; Papeo & Abassi, 2019; Vestner et al., 2019).

Automatic visual processing may also be limited to those relations that require little to no specialized world knowledge. The visual cues to the core force-dynamic relations we explored here—in and on—are quite general, involving (for example) patterns of occlusion or border ownership between two generic objects (Ullman et al., 2019) (although we should note that mere amount of occlusion of the Figure object was not sufficient to explain our results, as detailed in the analyses in the section titled Does Explicit Awareness of the Relational Categories Matter?). Indeed, the ease with which core notions of in and on are extracted perceptually may be what makes them so central to how human children and adults categorize and represent location in language (as discussed in the section titled From Language to Vision; Landau et al., 2016). By contrast, the “non-core” force-dynamic subtypes—e.g., a coat on (hanging from) a hook, or a stamp on (adhered to) an envelope—involve more specific knowledge about objects or non-intuitive physical forces (Landau, 2018). Thus, they may require more effort to be extracted perceptually, or may even be represented at a purely post-perceptual level.

### Relations for Intuitive Physics and Scene Understanding

The extraction of force-dynamic relations in automatic visual processing may also have implications for how observers intuit physical states of the world (e.g., what will move where; Kubricht et al., 2017; McCloskey et al., 1980; Ullman et al., 2017). Although some research suggests that such physical predictions are made via mental simulations that utilize a kind of “physics engine” in the head (e.g., Battaglia et al., 2013), other work proposes theoretical and empirical limits on such processes (e.g., Croom & Firestone, 2021; Davis & Marcus, 2015; Ludwin-Peery et al., 2019), leaving open how the mind accomplishes seemingly effortless inference about physical situations. Our work suggests that visual processing may automatically classify configurations of objects into abstract relational types (in or on)—perhaps even when the relations involve novel combinations of objects (Garnelo & Shanahan, 2019) or when the objects are totally unfamiliar or underspecified (Davis et al., 2017). Such categorizations could constrain or totally bypass more computationally intensive general-purpose simulation algorithms. For example, if containment is perceived, the mind may automatically infer that the contained object will move with its container, without having to actively simulate that outcome (Davis & Marcus, 2015). Future work may explore whether such physical contingencies are themselves elicited in a similarly automatic (and even visual) manner. Future work might also explore whether we encode unfolding physical scenes in terms of their *implied* relations, even when such relations are not yet instantiated (e.g., encoding an object falling *into* a container as in)—akin to representational momentum for visual events (Freyd, 1987; Hafri et al., 2022).

### Role-Filler Independence in Minds and Machines

Finally, our work may have implications for how visual processing should be modeled computationally and how it should be reproduced in machines. Recent artificial-intelligence systems using deep learning and other advanced neural-network architectures have achieved remarkable feats, recognizing objects at human levels (Krizhevsky et al., 2012; Yamins & DiCarlo, 2016) and even generating realistic images from text prompts (Ramesh et al., 2022; Saharia et al., 2022). However, systematic investigation of such models has revealed that they are unable to process many relations in a way that respects role-filler independence, including containment and support (Conwell & Ullman, 2022). For example, the recent text-guided image-generation model DALL-E 2 fails to accurately generate images for the seemingly simple prompt “a red cube on top of a blue cube”; instead, in many cases it reverses the roles of the cubes (i.e., blue-on-red) or generates just one cube with red and blue surfaces (Ramesh et al., 2022). We surmise that such systems may need to explicitly implement role-filler independence in their model architecture (or to implicitly discover how to do so) in order to overcome these gaps.

### Conclusions

The visual world is more than just a bag of objects; instead, objects are organized in ways that *relate* them to one another. Although perception research traditionally focuses on *what* features of objects we perceive (color, shape, motion), or *where* those objects are located in space, here we have explored how visual processing also encodes *how* those objects are arranged with respect to each other: The mind automatically extracts relations between objects, in ways that go beyond the objects themselves.

## ACKNOWLEDGMENTS

For helpful discussion and comments on earlier drafts, we acknowledge members of the Johns Hopkins University Perception & Mind Lab.

## FUNDING INFORMATION

This work was supported by the National Science Foundation under grant number BCS-2021053 awarded to C. F. and grant number SMA-2105228 awarded to A. H.

## DATA AVAILABILITY STATEMENT

All data and materials for the experiments (including preregistrations of Experiments 3a and 3b) are available at https://osf.io/nsd6z.

## AUTHOR CONTRIBUTION

A.H.: Conceptualization; Formal analysis; Funding acquisition; Investigation; Methodology; Resources; Software; Visualization; Writing – original draft; and Writing – review & editing. B.L.: Methodology; Project administration; Supervision; and Writing – review & editing. C.F.: Funding acquisition; Methodology; Project administration; Supervision; Writing – original draft; and Writing – review & editing. M.F.B.: Methodology; Project administration; Supervision; and Writing – review & editing.

## Notes

^1^ Throughout this paper, small caps (e.g. in) are used as shorthand for the relational representations under discussion. For example, in and on are used to indicate the mental representation of the relations containment and support, which are encoded in many languages using basic spatial terms (e.g., the prepositions “in” and “on” in English; Landau et al., 2016; Levinson & Wilkins, 2006).^2^ In all mixed-effects models reported in this paper, fixed effects of Epoch Number and Target Category were always included, unless stated otherwise. The random effects structure was the maximal structure that converged, starting with correlated random intercepts and slopes of Match Type and Target Category by participants and by target image’s object pair (Barr et al., 2013). When models did not converge, we simplified by first using uncorrelated intercepts and slopes, and followed that by dropping random slopes until convergence. Full details of random effects structures of models and model comparisons are available in the OSF repository.^3^ All results reported in this paper were qualitatively similar when performing simple paired *t*-tests on mean false-alarm rates by Match Type, across both participants and items (object pairs).^4^ This was also confirmed in a simple paired *t*-test on mean false-alarm rates across participants (*t*(168) = 3.54, *p* < .001, *d* = 0.27) and items (*t*(10) = 3.49, *p* = .006, *d* = 1.05).^5^ The difference between the two effects (same-relation and same-objects) may at first seem quite striking. However, same-object images share many more salient features beyond just (relatively abstract) object category—colors, textures, and sizes, among others—as compared to same-relation images. And indeed, results from our low-level control studies (Experiments 3a and 3b, discussed below) suggest that these low-level visual properties, rather than solely object categories, are the primary driver of object-confusion effects in the current task. In those control studies, images were distorted in ways that made objects difficult to recognize but preserved many low-level properties; yet same “object” false-alarm rates were actually *higher* in Experiments 3a and 3b (52.44% and 64.56%, respectively) than in Experiment 1 (41.67%)—even after accounting for the greater overall difficulty of the control studies, reflected by their higher different-object false-alarm rates (4.31% and 3.51% in Experiments 3a and 3b respectively, versus 1.23% in Experiment 1).^6^ As in Experiment 1, this was also confirmed in a simple paired *t*-test on mean false-alarm rates across participants (*t*(195) = 2.30, *p* = .022, *d* = 0.16) and items (*t*(10) = 3.10, *p* = .011, *d* = 0.93).^7^ While these continuous occlusion analyses may not explain the relational-confusion effects, the question remains open as to whether observers encoded images in terms of a *categorical* occlusion relation (and its absence), rather than in terms of in and on. This would still ultimately be interesting: occlusion is a relation (between three individuals: the Figure object, the Reference object, and the observer). And crucially, even in this scenario, we can speak to the main question of the paper: How are visual relations processed and represented? Our results suggest that the answer is: spontaneously, and in a format that respects role-filler independence. Ultimately, which visual relations fall under this umbrella is an empirical question, and we encourage further research here, using our case study as a roadmap for how to do so.^8^ These results were also confirmed by paired *t*-tests on mean false-alarm rates across participants and items. For Experiment 3a (diffeomorphs), by participants: *t*(126) = 0.07, *p* = .94, *d* = 0.006; and by items: *t*(10) = 0.07, *p* = .948, *d* = 0.02. For Experiment 3b (box-scrambling), by participants: *t*(123) = 1.41, *p* = 0.16, *d* = 0.13; and by items: *t*(10) = 0.75, *p* = .469, *d* = 0.23.^9^ It is unlikely that the lack of a relational-confusion effect in either control experiment was due to insufficient power. Although the greater difficulty of the target detection task in these control studies resulted in a higher number of exclusions than in the original study (16% exclusion in Experiment 1, versus approximately 37% exclusion in both Experiments 3a and 3b), we anticipated these exclusion rates from pilot data, and planned accordingly based on power analysis of the results from Experiment 1. This power analysis (conducted using the *simr* package in R) indicated that we would have approximately 92% power to detect a main effect of Match Type using the pre-registered mixed-effects logistic regression analyses.^10^ We can also compare the results between Experiments 1 and 2 (although we should qualify that the design of Experiment 2 was different from the others, since the alternate object-pair images did not appear in the latter). Even so, we found no significant difference between these experiments: a model with the interaction of Experiment and Match Type was not a significant improvement over a model without this interaction, *χ*^2^(1) = 2.05, *p* = .15.^11^ Note that a common empirical approach for probing the influence of language on perception or memory—linguistic interference tasks (e.g., verbal shadowing)—may not on its own resolve the issue here. Many such studies expect that linguistic interference tasks should *eliminate* the expected effects. By contrast, here we expect linguistic interference to have little to no effect, since we maintain that the locus of the relational-confusion effects is visual. Thus, if no effect of the interference task were observed, it would be unclear whether or not this was because the linguistic interference task was simply not powerful enough to entirely suppress linguistic encoding. Instead what would be needed is to also incorporate a *nonlinguistic* interference task equated in difficulty to the linguistic task (e.g., rhythm shadowing; Hermer-Vazquez et al., 1999; Trueswell & Papafragou, 2010). In our case, this nonlinguistic task would also have to be *visuospatial* (e.g., detecting changes in spatial grids overlaid on the scene; Endress & Potter, 2012), with the prediction that the nonlinguistic (but not linguistic) secondary task would eliminate the observed effects. Titrating the difficulty of the main and secondary tasks can be a challenge, however, so we leave this investigation for future work.
